# Exogenous Abscisic Acid Affects the Heat Tolerance of Rice Seedlings by Influencing the Accumulation of ROS

**DOI:** 10.3390/antiox12071404

**Published:** 2023-07-09

**Authors:** Yingfeng Wang, Bin Lei, Huabing Deng, Xiong Liu, Yating Dong, Wenjuan Chen, Xuedan Lu, Guihua Chen, Guilian Zhang, Wenbang Tang, Yunhua Xiao

**Affiliations:** 1 Hunan Provincial Key Laboratory of Rice and Rapeseed Breeding for Disease Resistance, College of Agronomy, Hunan Agricultural University, Changsha 410128, China; 2 State Key Laboratory of Hybrid Rice, Hunan Hybrid Rice Research Center, Changsha 410125, China; 3 National Center of Technology Innovation for Saline-Alkali Tolerant Rice, Hunan Hybrid Rice Research Center, Changsha 410125, China

**Keywords:** rice (*Oryza sativa* L.), seedling, ABA, heat stress, reactive oxygen species

## Abstract

Heat stress (HS) has become one of the major abiotic stresses that severely constrain rice growth. Abscisic acid (ABA) plays an important role in plant development and stress response. However, the effect of different concentrations of exogenous ABA on HS tolerance in rice still needs to be further elucidated. Here, we found that high concentrations of exogenous ABA increased HS damage in seedlings, whereas 10^−12^ M ABA treatment increased fresh and dry weight under HS relative to mock seedlings. Our further data showed that, in response to HS, 10^−5^ M, ABA-treated seedlings exhibited a lower chlorophyll content, as well as transcript levels of chlorophyll biosynthesis and antioxidant genes, and increased the accumulation of reactive oxygen species (ROS). In addition, the transcript abundance of some heat-, defense-, and ABA-related genes was downregulated on 10^−5^ M ABA-treated seedlings under HS. In conclusion, high concentrations of exogenous ABA reduced the HS tolerance of rice seedlings, and this negative effect could be achieved by regulating the accumulation of ROS, chlorophyll biosynthesis, and the transcription levels of key genes in seedlings under HS.

## 1. Introduction

Rice (*Oryza sativa* L.) is an important food crop that must face various biotic and abiotic stresses during its growth and development. Heat stress (HS) due to global warming is one of the most important factors that threaten rice yield. Under current rice cultivation patterns, climate warming could result in a 5.0% reduction in the total rice production in China by 2060 [1]. Rice is very sensitive to HS, and HS affects rice to varying degrees at all stages of its growth and development. HS in rice seedlings can cause growth retardation, reduced dry matter accumulation, leaf yellowing, and even seedling death [2,3,4]. Therefore, exploring the factors affecting HS tolerance in rice could provide an important and valuable reference for alleviating the food crisis in the context of global warming.

HS may lead to a range of physiological and biochemical changes in plants, such as protein denaturation, enzyme inactivation, and cell membrane damage [5]. In order to mitigate the effects of HS on their growth and development, higher plants have evolved many response mechanisms to cope with HS, including physiological and biochemical responses, signaling, gene expression, metabolite synthesis, etc. [6]. As with other abiotic stresses, HS also leads to the accumulation of reactive oxygen species (ROS), which can cause damage to a variety of cellular components and, thus, limit plant metabolic activities [7]. Plants counteract ROS accumulation caused by HS by synthesizing various enzymatic and non-enzymatic ROS scavenging and detoxification systems, of which enzymatic systems are usually considered to be the most effective forms [8].

As endogenous signaling molecules, plant hormones play an important role in regulating plant growth and development and defense processes [9]. Numerous studies in recent years have found that plant hormones are actively involved in the plant response to HS. Wassie et al. [10] showed that topical application of salicylic acid prior to HS improved morphological and physiological characteristics such as plant height, biomass, and the activities of antioxidant enzymes in alfalfa. Auxin mainly plays an important role in the plant morphological changes induced by HS, and *Arabidopsis* seedlings under HS promote hypocotyl elongation by increasing auxin concentration [11]. BRASSINAZOLE RESISTANT 1 (BZR1) is an important transcription factor of the brassinosteroid (BR) signaling pathway that regulates *FERONIA* (FER) homologs-mediated heat tolerance in tomatoes [12].

Abscisic acid (ABA) plays an important role as a stress hormone in the response of plants to HS. *Arabidopsis* mutants, defective in ABA signaling and ABA biosynthesis, are sensitive to HS, and the overexpression of the ABA-responsive element-binding protein (AREB) enhances HS tolerance [13]. ABA is mainly involved in plant responses to HS by mediating their antioxidant capacity, heat shock proteins (HSPs), and sugar metabolism [14]. ABA synthesis-deficient mutants or the exogenous application of an ABA synthesis inhibitor treatment can change ABA and H_2_O_2_ levels and weaken the HS tolerance of plants [15]. HS can rapidly induce the accumulation of HSPs, which mediate the homeostasis of cells under HS by helping proteins to re-establish their normal conformation. In maize, ABA induces the post-transcriptional regulation of sHSP17.2, sHSP17.4, and sHSP26 expression in the leaves [16]. It has been shown that the crosstalk between ABA and HSP70 can protect proteins and enzymes from degradation at high temperatures [17,18]. In addition, ABA has been found to regulate carbohydrate and energy status by affecting the transport and metabolic levels of sucrose, thereby enhancing plant HS tolerance [14].

In summary, ABA is widely involved in the regulation of plant HS response and also plays an important regulatory role in several processes of HS. Studies on the relationship between exogenous ABA and plant HS tolerance have been conducted by spraying, and these effects have been positive [19,20]. However, the effect of different concentrations of exogenous ABA on the heat tolerance of rice seedlings remains unclear. Therefore, in this study, we investigated the mechanism for the differential regulatory effects of different concentrations of exogenous ABA on HS tolerance in rice by means of hydroponic experiments. This study could further elucidate the role of ABA in mediating plant HS responses and provide more references for studying the relationship between ABA and plant HS.

## 2. Materials and Methods

### 2.1. Plant Materials and HS Treatment

The *japonica* rice variety Zhonghua 11 was used as a research object. Rice plants were grown hydroponically in a culture chamber at a temperature of 28 °C during the day and 28 °C at night, with 12 h of day/night, 70% humidity, and a light intensity of 540 μmol·m^−2^·s^−1^. The Kimura B nutrient solution (pH 5.6–5.8) provides nutrients to seedlings [21].

Rice seedlings cultured for 10 days were used for 45 °C HS treatment. Currently, ABA transporters have been identified in a variety of plants, such as ABCG25, ABCG40, DTX50, AIT1-4, SlAIT1.1, DG1, and OsPM1, which are involved in the uptake and in vivo transport of ABA [22]. Meanwhile, it has been reported that the application of exogenous ABA in the solution can increase the ABA content in plants [23,24]. In this study, exogenous ABA was applied to the hydroponic nutrient solution, and three groups of ABA concentrations were set up, with exogenous ABA concentrations of 10^−13^, 10^−12^, and 10^−11^ M as a low concentration ABA treatment group, exogenous ABA concentrations of 10^−10^, 10^−9^, and 10^−8^ M as a medium concentration ABA treatment group, and exogenous ABA concentrations of 10^−7^, 10^−6^, and 10^−5^ M as a high concentration ABA treatment group. Control plants (mock) without exogenous ABA were set up for each group.

The medium and low concentration ABA treatment groups were treated at 45 °C for 36 h and the high concentration ABA treatment group was treated at 45 °C for 35 h and then recovered at 28 °C for 7 days, followed by phenotypic photography and the measurement of shoot dry and fresh weights.

Treatments with exogenous ABA concentrations of 10^−5^ M and 10^−12^ M were selected for further experiments. The mock, 10^−5^ M ABA-treated, and 10^−12^ M ABA-treated seedlings were subjected to 45 °C for 30 h in a culture chamber for HS treatment or were grown at 28 °C for 30 h in another culture chamber to be used as the control. Samples of the treated and control plants were collected for the determination of relevant indexes.

### 2.2. Fresh and Dry Weights of Shoot

Seven days after the recovery of the control conditions or HS-treated seedlings, the fresh weight of the seedlings in each treatment was measured, and these seedlings were subsequently baked at 105 °C for 15 min and then baked at 80 °C to a constant weight and the dry weight was measured. There were three replicates of each treatment, and each replicate included eight seedlings.

### 2.3. Chlorophyll and Carotenoid Content

Chlorophylls and the carotenoid were extracted from 0.1 g of fresh rice leaves using 96% ethanol. The absorbance of the extract was recorded at 665, 649, and 470 nm, and the chlorophyll and carotenoid contents were calculated according to the method of Lichtenthaler and Wellburn [25].

### 2.4. ROS Accumulation and Electrolyte Leakage

Rice seedlings were exposed to HS (45 °C) for 30 h. Seedlings grown under normal conditions in another growth chamber were used as the controls. After treatment, leaf samples were immediately collected to measure the accumulation of ROS and electrolyte leakage. Nitro blue tetrazolium (NBT) and 3,3’-diaminobenzidine (DAB) staining methods were used to detect the accumulation of O_2_^•−^ and H_2_O_2_ in the leaves, as previously described [26]. O_2_^•−^ and H_2_O_2_ were measured using a kit (BC1290 and BC3595, Beijing, China) according to the manufacturer’s protocol. Briefly, the extracting solution of O_2_^•−^ was reacted with hydroxylamine hydrochloride to produce NO_2_^−^, and NO_2_^−^ was reacted with aminobenzenesulfonamide and *N*-1-Naphthylethylenediamine dihydrochloride to form a purple-red azo compound, and the absorbance of the reaction mixture was measured at 530 nm. The H_2_O_2_ content was measured by reacting the extract with titanium sulfate to form a yellow titanium peroxide complex and then measuring the absorbance at 410 nm. Electrolyte leakage in the leaves of each treated plant was detected according to the previously described method [26].

### 2.5. Quantitative Real-Time PCR

A quantitative Real-Time PCR (qRT-PCR) was used to detect the transcript levels of some genes in the control and HS-treated leaves. The total RNA extraction, reverse transcription, and qRT-PCR assays were performed as described previously [27]. Rice *OsActin1* was used as an internal reference gene, and the relative transcript levels were calculated as we described [28]. The gene expression was quantified using the 2^−ΔΔCT^ method. Three biological replicates were applied throughout the assay. The primer sequences used for qRT-PCR are shown in Supplemental Table S1.

### 2.6. Statistical Analysis

All results are presented as the mean ± SD (*n* = 3). Statistical analyses were performed using DPS (v7.05) and plotted using GraphPad Prism (v8.0.2). One-way ANOVA was used to statistically analyze the data, and differences were considered significant at *p* < 0.05 by Duncan’s multiple range test.

## 3. Results

### 3.1. Effect of Exogenous ABA Application on HS Tolerance in Seedlings

**Figure 1:**
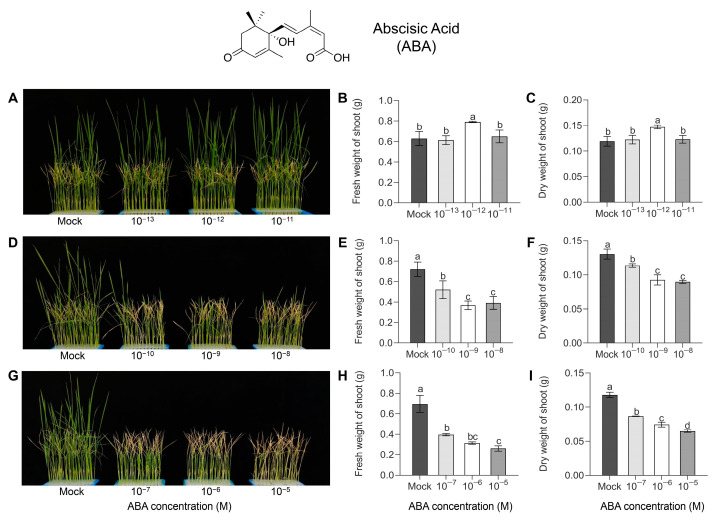
Effect of exogenous application of different concentrations of ABA on HS tolerance of seedlings. Recovery of 7-day phenotype of seedlings after HS treatment at 45 °C under the exogenous application of low (**A**), medium (**D**), and high (**G**) concentrations of ABA. Statistical data of fresh (**B**,**E**,**H**) and dry (**C**,**F**,**I**) weights of seedlings in (**A**,**D**,**G**), respectively. Data are expressed as mean ± SD. Different letters indicate significant differences by one-way ANOVA test (*n* = 3, *p* < 0.05).

To investigate the effect of exogenous ABA on HS tolerance in rice, we measured the fresh and dry weights of the rice seedlings treated with different concentrations of an exogenous ABA applied under HS. For the low-concentration ABA treatment group, we found no significant differences in the fresh and dry weight of plants treated with 10^−13^ and 10^−11^ M ABA compared to mock seedlings after HS treatment and recovery for 7 days, while these were significantly higher in plants treated with 10^−12^ M ABA compared to mock seedlings, with a 23.5% increase in the dry weight (Figure 1A–C). By contrast, after HS treatment and recovery for 7 days, there was a significant decrease in the fresh and dry weight of seedlings with medium (10^−10^, 10^−9^, and 10^−8^ M) and high (10^−7^, 10^−6^, and 10^−5^ M) concentrations of exogenous ABA applied to relative to mock seedlings (Figure 1D–I). However, under control conditions, exogenous ABA treatment had no significant effect on the fresh and dry weight of seedlings compared to the respective mock plants (Supplementary Figure S1). These results indicate that the application of high and medium concentrations of exogenous ABA significantly reduced the HS tolerance of seedlings.

### 3.2. High Concentration of Exogenous ABA Decreased Chlorophyll Content in Seedling under HS

**Figure 2:**
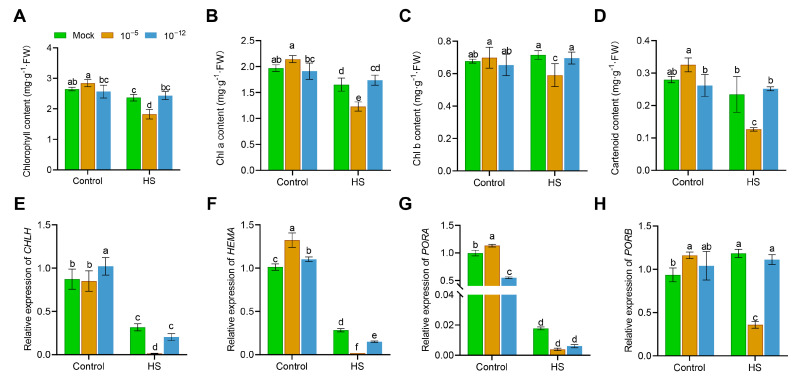
Effect of the exogenous application of 10^−5^ M and 10^−12^ M ABA on chlorophyll content and chlorophyll biosynthesis genes in seedlings under HS. The contents of chlorophyll (**A**), chlorophyll a (chl a; **B**), chlorophyll b (chl b; **C**), and carotenoid (**D**) in mock, 10^−5^ M ABA-treated, and 10^−12^ M ABA-treated seedling leaves under control conditions or 45 °C HS treatment for 30 h. Transcript levels of *CHLH* (**E**), *HEMA* (**F**), *PORA* (**G**), and *PORB* (**H**) in mock, 10^−5^ M ABA-treated, and 10^−12^ M ABA-treated seedlings under control conditions or 45 °C HS treatment for 30 h. Data are expressed as the mean ± SD. Different letters indicate significant differences by the one-way ANOVA test (*n* = 3, *p* < 0.05).

To reveal the possible mechanisms by which exogenous ABA affected HS tolerance in rice, we selected two concentrations with positive (10^−12^ M) and negative (10^−5^ M) effects on seedling HS tolerance for further study. We first measured the chlorophyll, chlorophyll a (chl a) and chlorophyll b (chl b), and carotenoid contents of the control conditions and HS-treated seedlings. As shown in Figure 2A–D, after 30 h of HS at 45 °C, the chlorophyll content, as well as chl a, ch b, and the carotenoid content of the seedlings exogenously applied with 10^−5^ M ABA, were significantly lower than those of the mock seedlings. However, 10^−12^ M ABA-treated seedlings showed no significant difference compared to the mock seedlings after HS (Figure 2A–D).

Next, we used qRT-PCR assays to examine the transcript levels of four key chlorophyll biosynthetic genes in the leaves of seedlings from each treatment. As shown in Figure 2E–H, the transcript levels of *CHLH*, *HEMA*, *PORA*, and *PORB* in 10^−5^ M ABA-treated seedlings were significantly downregulated under HS compared to those in the mock seedlings. However, no clear differences in these chlorophyll biosynthesis genes were observed between the mock and 10^−12^ M ABA-treated seedlings under HS, except for *HEMA* (Figure 2E–H). These data suggest that the exogenous application of 10^−5^ M ABA had a negative effect on the chlorophyll content and chlorophyll biosynthesis gene transcription in seedlings under HS.

### 3.3. High Concentration of Exogenous ABA Increased the Accumulation of ROS in Seedlings under HS

**Figure 3:**
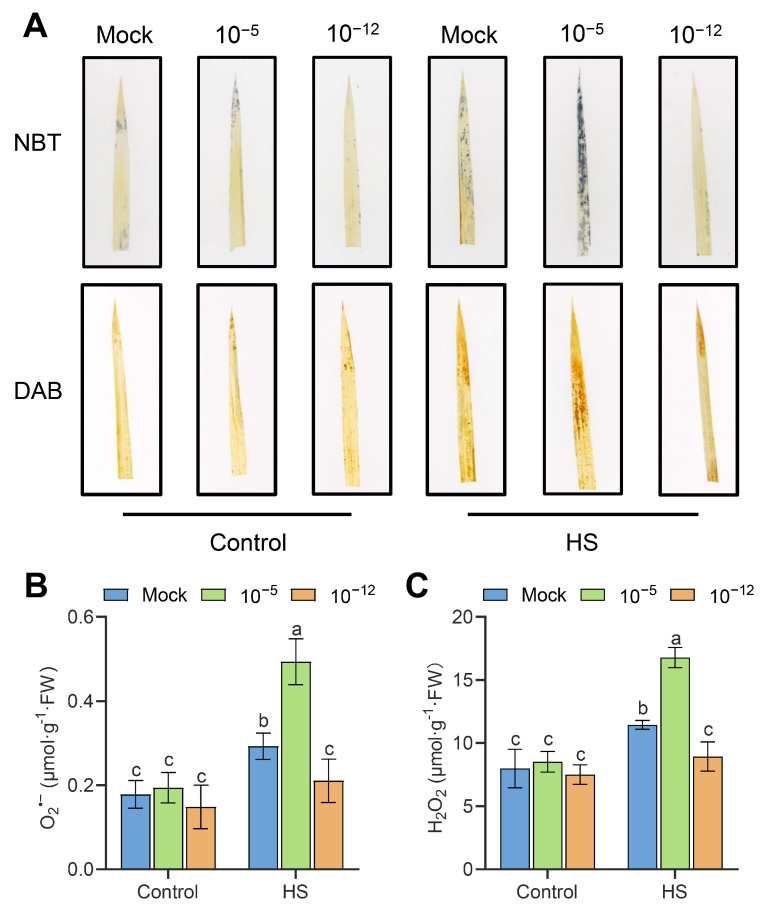
O_2_^•−^ and H_2_O_2_ levels in the mock, 10^−5^ M ABA-treated, and 10^−12^ M ABA-treated seedlings under HS. (**A**) NBT and DAB staining of the leaves of mock, 10^−5^ M ABA-treated, and 10^−12^ M ABA-treated seedlings upon HS. (**B**) O_2_^•−^ content in mock, 10^−5^ M ABA-treated, and 10^−12^ M ABA-treated seedlings after 30 h of 45 °C treatment. (**C**) Quantitative measurement of total H_2_O_2_ content in mock, 10^−5^ M ABA-treated, and 10^−12^ M ABA-treated seedlings under HS or control conditions. Data are expressed as mean ± SD. Different letters indicate significant differences by one-way ANOVA test (*n* = 3, *p* < 0.05).

To test the ROS scavenging activity of exogenous ABA on seedlings under HS, we exposed mock, 10^−5^ M ABA-treated, and 10^−12^ M ABA-treated seedlings to HS (45 °C) for 30 h and stained their leaves for NBT or DAB. As shown in Figure 3A, there were no significant differences in NBT staining (brown pigment) and DAB staining (blue pigment) between the leaves of mock, 10^−5^ M ABA treated, and 10^−12^ M ABA treated leaves under the control conditions. By contrast, under HS treatment, the leaves of seedlings treated with 10^−5^ M ABA stained darker compared to the mock seedlings, whereas the leaves of 10^−12^ M ABA-treated seedlings stained lighter (Figure 3A). Furthermore, we further quantified the differences in the O_2_^•−^ and H_2_O_2_ content between the mock, 10^−5^ M ABA-treated, and 10^−12^ M ABA-treated seedlings under HS or control conditions. As shown in Figure 3B,C, there was no significant difference in the O_2_^•−^ or H_2_O_2_ content between the mock, 10^−5^ M ABA-treated, and 10^−12^ M ABA-treated seedlings under control conditions. However, consistent with the staining results, 10^−5^ M ABA-treated seedlings had a higher, while 10^−12^ M ABA-treated seedlings had a lower O_2_^•−^ and H_2_O_2_ content than the mock seedlings when exposed to 30 h of HS (Figure 3B,C).

Next, we measured the electrolyte leakage rate of the seedlings in each treatment under the control and HS. As shown in Supplementary Figure S2, there was no significant difference in the electrolyte leakage rate between mock, 10^−5^ M ABA-treated, and 10^−12^ M ABA-treated seedlings under control conditions. By contrast, after HS, electrolyte leakage was significantly higher in 10^−5^ M ABA-treated seedlings than in mock and 10^−12^ M ABA-treated seedlings (Supplementary Figure S2). These results indicate that the exogenous application of 10^−5^ M ABA increased ROS accumulation and oxidative damage in seedlings under HS.

### 3.4. Transcriptional Changes of Antioxidant Enzyme Genes in Exogenous ABA-Treated Seedlings under HS

**Figure 4:**
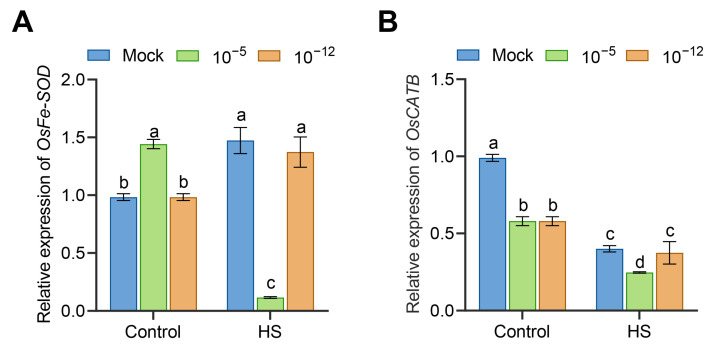
Transcriptional expression of *OsFe-SOD* (**A**) and *OsCATB* (**B**) in mock, 10^−5^ M ABA-treated, and 10^−12^ M ABA-treated seedlings under control conditions or HS, as detected by qRT-PCR. Data are expressed as mean ± SD. Different letters indicate significant differences by one-way ANOVA test (*n* = 3, *p* < 0.05).

To further characterize the effect of exogenous ABA on the antioxidant enzymes in seedlings under HS, we determined the relative expression of two antioxidant enzyme genes, *OsFe-SOD* and *OsCATB*. Under the control conditions, the transcript levels of *OsFe-SOD* were significantly higher in 10^−5^ M ABA-treated seedlings compared to mock seedlings, and the transcripts of *OsCATB* were significantly less in the two ABA concentrations-treated seedlings than in the mock seedlings (Figure 4A,B). By contrast, under HS, the expressions of both *OsFe-SOD* and *OsCATB* were significantly down-regulated in 10^−5^ M ABA-treated seedlings compared with the mock seedlings; however, these expressions were not significantly different in 10^−12^ M ABA-treated seedlings (Figure 4A,B). These data suggest that the 10^−5^ M ABA treatment had a negative effect on the expression of antioxidant enzyme-related genes under HS.

### 3.5. Transcriptional Changes of ABA-Responsive Genes in Exogenous ABA-Treated Seedlings under HS

**Figure 5:**
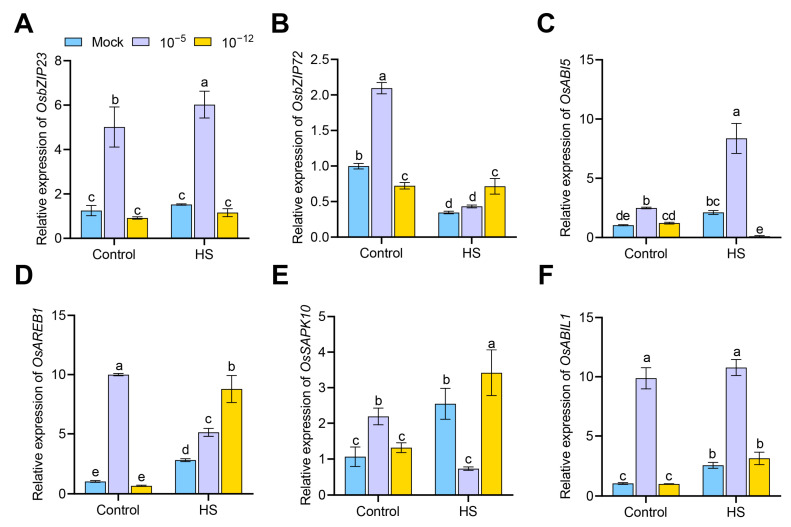
Transcriptional expression of ABA-responsive genes (*OsbZIP23*; (**A**), *OsbZIP72*; (**B**), *OsABI5*; (**C**), *OsAREB1*; (**D**), *OsSAPK10*; (**E**), and *OsABIL1*; (**F**) in mock, 10^−5^ M ABA-treated, and 10^−12^ M ABA-treated seedlings under control conditions or HS, as detected by qRT-PCR. Data are expressed as mean ± SD. Different letters indicate significant differences by one-way ANOVA test (*n* = 3, *p* < 0.05).

Our results suggest that exogenous ABA altered HS tolerance in the rice seedlings, which prompted us to examine the transcriptional changes of ABA-responsive genes in each treated seedling upon HS. These ABA-responsive genes included four genes encoding the bZIP transcription factor (*OsbZIP23*, *OsbZIP72*, *OsABI5*, and *OsAREB1*), one gene encoding SnRK2 protein kinase (*OsSAPK10*), and one gene encoding clade A type 2C protein phosphatase (*OsABIL1*). As shown in Figure 5A–F, the transcript levels of all six genes were up-regulated by the 10^−5^ M ABA treatment under control conditions. After HS treatment, the transcription of *OsbZIP23*, *OsABI5*, *OsAREB1*, and *OsABIL1* was significantly promoted; however, the transcription of *OsSAPK10* was significantly inhibited in 10^−5^ M ABA-treated seedlings compared with the mock seedlings (Figure 5A,C–F). However, the transcript of *OsbZIP72*, *OsAREB1*, and *OsSAPK10* was significantly up-regulated. By contrast, the transcription of *OsABI5* was significantly inhibited in 10^−12^ M ABA-treated seedlings compared with the mock seedlings under HS (Figure 5B–E).

### 3.6. Transcriptional Changes of Heat- and Defense-Related Genes in Exogenous ABA-Treated Seedlings under HS

**Figure 6:**
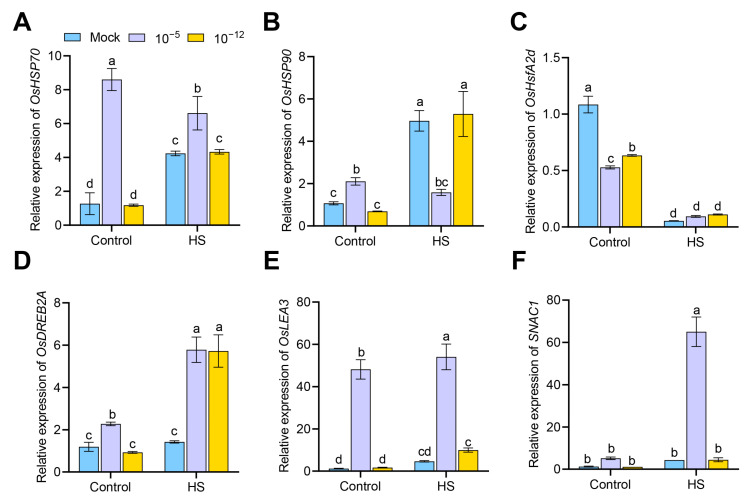
Transcriptional expression of heat-related genes (*OsHSP70*; (**A**), *OsHSP90*; (**B**), and *OsHsfA2d*; (**C**) and stress defense genes (*OsDREB2A*; (**D**), *OsLEA3*; (**E**), and *SNAC1*; (**F**) in mock, 10^−5^ M ABA-treated, and 10^−12^ M ABA-treated seedlings under control conditions or HS, as detected by qRT-PCR. Data are expressed as mean ± SD. Different letters indicate significant differences by one-way ANOVA test (*n* = 3, *p* < 0.05).

To elucidate the possible molecular mechanisms by which exogenous ABA affected HS tolerance, we used qRT-PCR to examine the transcript levels of several representative heat- and defense-related genes in mock, 10^−5^ M ABA-treated and 10^−12^ M ABA-treated seedlings under either the control or HS conditions. These genes included three heat-related genes (two shock protein genes, *OsHSP70* and *OsHSP90*, and the heat shock transcription factor gene *OsHsfA2d*) and three stress defense genes (AP2/EREBP transcription factor gene *OsDREB2A*, late embryonic enrichment protein gene *OsLEA3*, and stress-responsive NAC transcription factor gene *SNAC1*). As shown in Figure 6A–F, under the control conditions, the 10^−5^ M ABA treatment up-regulated the transcript levels of *OsHSP70*, *OsHSP90*, *OsDREB2A*, *OsLEA3*, and *SNAC1*, but both the 10^−12^ and 10^−5^ M ABA treatment down-regulated the transcript level of *OsHsfA2d* compared with the mock. Under HS, the 10^−5^ M ABA treatment up-regulated the transcript levels of *OsHSP70*, *OsDREB2A*, *OsLEA3*, and *SNAC1* but down-regulated the transcript level of *OsHSP90* compared with the mock (Figure 6A–F). However, 10^−12^ M ABA treatment only up-regulated the transcript level of *OsDREB2A* compared with the mock (Figure 6D). These results suggest that ABA treatment regulated the expression of heat- and defense-related genes to affect HS tolerance.

## 4. Discussion

With the increase in extremely hot weather, HS has widely affected rice production; therefore, there is an urgent need to find effective methods to improve the HS tolerance of rice. It has been demonstrated that ABA plays an important role in HS in plants [14]. However, current knowledge of how exogenous ABA regulates HS tolerance in rice seedlings is still very limited. In this study, we reported the physiological and molecular responses of rice seedlings with the application of different exogenous ABA concentrations under HS. Our results demonstrate that the application of high concentrations of exogenous ABA (10^−5^ M) can reduce HS tolerance in rice seedlings by affecting their ROS accumulation and chlorophyll content as well as the expression of chlorophyll biosynthetic-, ABA- and heat-, and defense-related genes.

In recent years, numerous studies have found that the application of exogenous plant hormones, including ABA, can positively improve the HS tolerance of plants [9]. ABA spraying at the flowering stage enhances the seed-setting rate in heat-stressed rice by affecting trehalose metabolism and ATP consumption [20]. Exogenous ABA pretreatment greatly improved the scavenge ability of ROS, thereby reducing leaf apoptosis and chlorophyll loss in rice seedlings [29]. Notably, in our study, we observed that ABA negatively regulated HS tolerance in the seedlings when the exogenous ABA concentration was too high (Figure 1A–F). By contrast, the fresh and dry weights of seedlings were significantly higher than those of the mock seedlings only with 10^−12^ M ABA treatment (Figure 1G–I). These data suggest that the effect of exogenous ABA on HS tolerance in rice is twofold, and excessive exogenous ABA concentrations can reduce HS tolerance in rice seedlings.

The massive accumulation of ROS caused by HS leads to oxidative damage to the membranes and protein denaturation, and even cell death [30]. Therefore, the strict control of ROS levels is essential for plants to resist HS damage. It has been widely demonstrated that ABA confers abiotic stress resistance to plants by regulating ROS homeostasis. Endogenous ABA increases antioxidant activity through the modulation of the *OsSAPK9*-*OsbZIP20* pathway, thereby reducing the accumulation of ROS caused by ammonium toxicity [31]. *ABI4* is a key component of ABA signaling that mediates ROS metabolism by directly combining with and inducing the expression of RbohD [32]. In the present study, 10^−5^ M ABA-treated seedlings exhibited more ROS accumulation and lower transcript levels of *OsFe-SOD* and *OsCATB* than the mock and 10^−12^ M ABA-treated seedlings under HS (Figure 3 and Figure 4). Meanwhile, 10^−12^ M ABA-treated seedlings accumulated less O_2_^•−^ and H_2_O_2_ content under HS (Figure 3). Consistent with our conclusion, the ROS content in developing anthers increased significantly at HS under the higher exogenous ABA incubation compared with the control (no ABA addition), whereas no significant changes were observed under lower exogenous ABA incubation [33]. Therefore, higher concentrations of exogenous ABA could reduce HS tolerance to the seedlings by weakening their antioxidant capacity, leading to the accumulation of ROS and membrane damage.

HS affects chloroplast development and activity, as well as plant photosynthesis. Rice *white panicle2* (*wp2*) mutants caused by mutations in the *OsTRXz* gene in rice chloroplasts exhibited a heat-sensitive phenotype and reduced the editing efficiency of chloroplast genes under HS conditions [34]. The rice *high temperature enhanced lesion spots 1* (*hes1*) mutant, under HS, resulted in a higher accumulation of ROS and more severe chloroplast degradation [35]. In our study, 10^−12^ M ABA could maintain the chlorophyll content and transcript levels of chlorophyll biosynthetic genes in seedlings under HS comparable to those of mock plants (Figure 2). However, 10^−5^ M ABA-treated seedlings exhibited a lower chlorophyll content and expression of chlorophyll biosynthetic genes at HS (Figure 2). We speculated that this reduction in chlorophyll synthesis in seedlings under HS due to high ABA concentration might be partly responsible for the reduced HS tolerance of seedlings. To support this inference, Yang et al. [36] showed that At-NAP induces the expression of the ABA biosynthesis gene *AAO3* by binding to its promoter, which further promotes chlorophyll degradation and elevated ABA levels in *Arabidopsis*, while elevated ABA, in turn, induces the expression of the *NAP* gene.

Plant hormones can have opposite effects on plant growth and development at different concentrations or tissues, and accordingly, plants may respond to different concentrations of plant hormones through different signaling pathways [37]. LIC and BZR1 act as antagonistic effects in response to high and low brassinosteroids, respectively, to balance the BR signal and control leaf bending in rice [38]. In the present study, we examined transcriptional changes in a number of ABA- and heat-, defense-, related genes that had positive effects on plant stress resistance (Figure 5 and Figure 6) [39,40,41] It should be emphasized that under HS, not all of these genes were transcribed at lower levels in 10^−5^ M ABA-treated seedlings than in 10^−12^ M ABA-treated seedlings (Figure 5 and Figure 6). The signaling pathways leading to opposite HS tolerance at different concentrations of exogenous ABA are complex and identifying their key regulatory genes can be challenging. Our results provide some clues about available genes, such as *OsHSP90*, *OsbZIP72*, *OsAREB1, OsSAPK10*, etc. The overexpression of *OsbZIP72* in rice improves resistance to oxidative stress and tolerance to drought and salinity stress [42]. The co-expression of *OsbZIP46* (*OsAREB1*) and *SAPK6* enhances resistance to HS in rice [43]. In subsequent studies, the further dissection of the exact role of these genes in response to different concentrations of ABA and HS tolerance is required using ABA- or heat-related mutants.

## 5. Conclusions

Our study reveals the effect of the exogenous application of different concentrations of ABA on HS tolerance in rice seedlings. After high-temperature stress and recovery for 7 days, high exogenous ABA treatment significantly reduced the fresh and dry weights of rice seedlings, while only 10^−12^ M ABA-treated seedlings had higher fresh and dry weights than the mock plants. Further studies revealed how high concentrations (10^−5^ M) of exogenous ABA exhibited higher ROS accumulation, while the chlorophyll content and transcript levels of chlorophyll biosynthesis- and antioxidant enzyme-related genes were lower. In summary, the effect of exogenous ABA on the HS tolerance of rice seedlings is twofold, and the concentration of exogenous ABA is crucial in changing the HS tolerance of rice seedlings.

## Data Availability

All of the data is contained within the article and the supplementary materials.

## Supplementary Materials

The following supporting information can be downloaded at: https://www.mdpi.com/article/10.3390/antiox12071404/s1, Table S1: The list of RT-qPCR primer pairs; Figure S1: Effect of exogenous application of different concentrations of ABA on the fresh and dry weight of seedlings under controlled conditions; Figure S2: Electrolyte leakage rates in mock, 10^−5^ M ABA-treated, and 10^−12^ M ABA-treated seedlings after 30 h of 45°C treatment.
