# Supplementary material for: Exogenous Abscisic Acid Affects the Heat Tolerance of Rice Seedlings by Influencing the Accumulation of ROS
Source: Antioxidants (Basel). 2023 Jul 9;12(7):1404. doi: 10.3390/antiox12071404 (PMC10376659; doi:10.3390/antiox12071404)
Supplement: Supplementary file 1 [file antioxidants-12-01404-s001.zip › antioxidants-2431449-supplementary.pdf]

Table S1. The list of RT-qPCR primer pairs.

Primer name	Sequence (5'–3')	MSU_Locus
OsACTIN1-F	GACCTTCAACACCCCTGCTA	LOC_Os03g50885
OsACTIN1-R	GAGTCCAACACAATACCTGTGG	
CHLH-F	AACTGGATGAGCCAGAAGAGA	LOC_Os03g20700
CHLH-R	AAATGCAAAAGACTTGCGACT	
HEMA-F	CGCTATTTCTGATGCTATGGGT	LOC_Os10g35840
HEMA-R	TCTTGGGTGATGATTGTTTGG	
PORA-F	ATGGCTCTCCAAGTTCAG	LOC_Os04g58200
PORA-R	TGGCTCACGCTAAGGAAC	
PORB-F	CCGCAAGGAGGGAGCGGTG	LOC_Os10g35370
PORB-R	CCCTCTTGGTGCTAAGGCCG	
OsFe-SOD-F	CTTGATGCCCTGGAACCTTA	LOC_Os06g05110
OsFe-SOD-R	GCCAGACCCCAAAAGTGATA	
OsCATB-F	GTTCGGTTCTCCACAGTCGT	LOC_Os06g51150
OsCATB-R	CCCTCCATGTGCCTGTAGTT	
OsHSP70-F	GCCAAGCGTCAAGCAGTGACCAA	LOC_Os03g02260
OsHSP70-R	GGTCATCAAAGCGCCGCCCTAT	
OsHSP90-F	TTTGGGCGAAGGTGACACTGCTA	LOC_Os06g50300
OsHSP90-R	TGGCAATGGTCCCAAGGTTCTTAAT	
OsHsfA2d-F	CAAGAGATGATGCTGGGATTCC	LOC_Os03g06630
OsHsfA2d-R	CTATTGCTTAGATAACCCAGCT	
OsDREB2A-F	GGAATCTCCTCCTTTCATCGTG	LOC_Os01g07120
OsDREB2A-R	TTCCGCTCCTGACAAACACG	
OsLEA3- F	GCAGTACACCAAGGACTCTGC	LOC_Os05g46480
OsLEA3- R	ACCCAAAGGGAAATCATTCAC	
SNAC1-F	CATGGTCCCGTTCTGAGGTG	LOC_Os03g60080
SNAC1-R	CACACGTTGCAGCATCGATC	
OsbZIP23-F	GGAGCTGAACGATGAACTCCAG	LOC_Os02g52780
OsbZIP23-R	TCGGCTCATTCTCTCTAGAACCTC	
OsbZIP72-F	AAGAGACTTTGCTTGCGAAGAAC	LOC_Os09g28310
OsbZIP72-R	TGATGAAGGTGGGAATTTGCA	
OsABI5- F	GAAGAATTCCAGGCTACCACC	LOC_Os01g64000
OsABI5- R	TTGATCTCAGTCCACACCTCC	
OsAREB1-F	TGAGGTGGCAAAACTGAAGGA	LOC_Os06g10880
OsAREB1-R	CTGTATCATTTCCACCTGCTTTTTC	
SAPK10-F	TCTTGACGTGGAAAGCAGTG	LOC_Os03g41460
SAPK10-R	TCCACTTCCCAGTTTCTTGG	
OsABIL1-F	GATGGTCTCTGGGACGTCAT	LOC_Os01g40094
OsABIL1-R	CTTCGACAAGCACTCAGCAG	

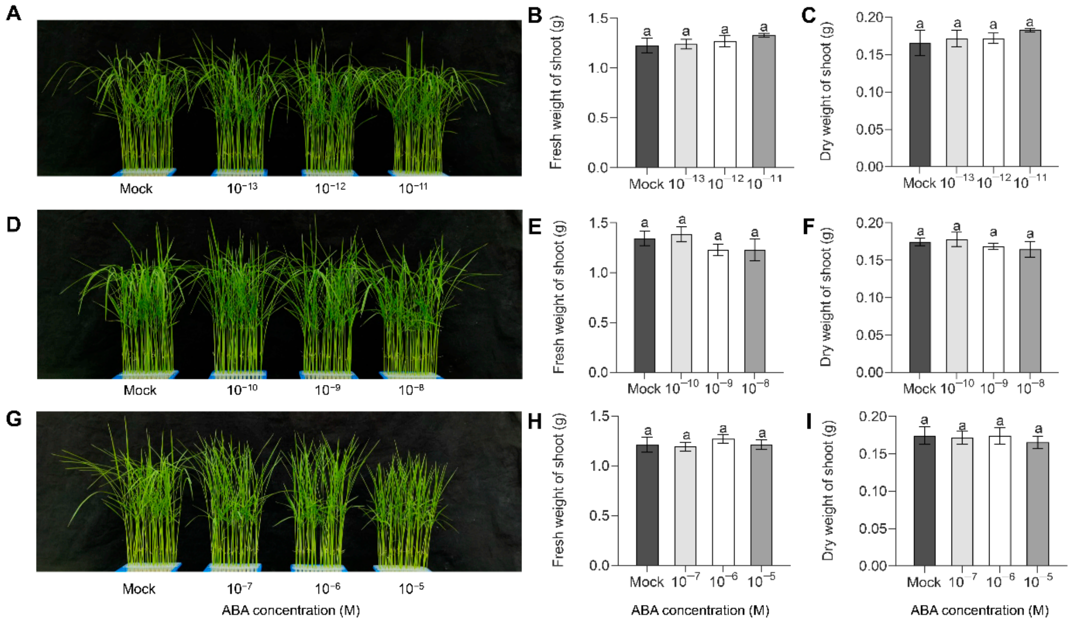

Figure S1. Effect of exogenous application of different concentrations of ABA on the fresh and dry weight of seedlings under controlled conditions.

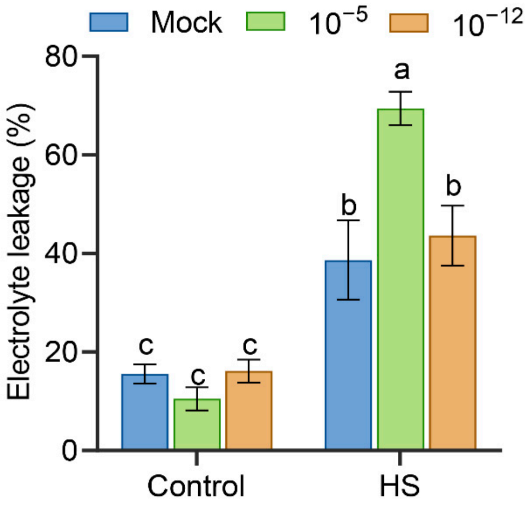

Figure S2. Electrolyte leakage rates in mock, 10^{-5} M ABA-treated, and 10^{-12} M ABA-treated seedlings after 30 h of 45°C treatment.
